# Preparation and Properties of Epoxy Modified Acrylic Polymer

**DOI:** 10.3390/polym17030380

**Published:** 2025-01-30

**Authors:** Shiyan Zhou, Jinmei Ma, Jun-Wen Yu, Zhigang Gao, Fei Li, Fenghua Zhang, Yu-Peng He

**Affiliations:** 1 School of Petrochemical Engineering, Liaoning Petrochemical University, No. 1 Dandong West Road, Fushun 110031, China; shiyanzhou925@163.com (S.Z.); majinmei_21@163.com (J.M.); lifei2008@lnpu.edu.cn (F.L.); 2 State Key Laboratory of Fine Chemicals, Ningbo Institute of Dalian University of Technology, Ningbo 315016, China; 15105844508@163.com (J.-W.Y.); gzg1980@dlut.edu.cn (Z.G.); 3 College of Materials Science and Engineering, Shenyang University of Chemical Technology, No. 11 Street, Economic and Technological Development Zone, Shenyang 110142, China; 4 School of Chemical Engineering, Dalian University of Technology, No. 2 Linggong Road, Dalian 116024, China

**Keywords:** epoxy acrylate viscosity reducer, heavy oil reduces viscosity, viscosity reduction

## Abstract

This paper describes the synthesis of a viscosity-reducing agent using butyl acrylate (BA), ethyl methacrylate (EMA), acrylic acid (AA) and *N*-hydroxymethylacrylamide (N-MAM) monomers through emulsion polymerization. A series of viscosity-reducing agents were developed by incorporating varying amounts of glycidyl methacrylate (GMA) monomers. The reaction mechanism of epoxy acrylate viscosity reducer was analyzed by Fourier transform infrared spectroscopy (FTIR). Additionally, the particle size and Zeta potential were used to analyze the stability of the polymer and the difference in the polymer after adding GMA monomer. Thermogravimetric (TG) analysis indicated a significant improvement in the thermal stability of the resin due to GMA modification. The viscosity reduction test results demonstrated a substantial decrease in the viscosity of heavy oil, along with a notable increase in the viscosity reduction rate. The FTIR analysis results confirmed that GMA successfully introduced polyacrylate molecular chains. Furthermore, particle size and Zeta potential measurements showed that the average particle size of the emulsion increased from 132 nm to 187 nm, while the Zeta potential changed from −43 mV to −40 mV with the addition of 15% GMA. Compared with W0, the final thermal degradation temperature of W15 increased from 450 °C to 517 °C. When the GMA content reached 15 wt%, the maximum weight loss temperature increased by approximately 12 °C compared to the sample without GMA. Specifically, adding 8% W15 epoxy acrylate resulted in an 89% viscosity reduction rate for heavy oil, demonstrating an excellent viscosity reduction effect. This study successfully developed a novel epoxy acrylate viscosity reducer using a simple synthesis method, showcasing excellent stability, cost-effectiveness and remarkable viscosity reduction.

## 1. Introduction

The increasing demand for energy and the continuous consumption of thin oil resources have led to extensive attention on the exploitation and use of heavy oil [1,2,3]. One common method to reduce the viscosity of heavy oil is by forming an oil-in-water (O/W) emulsion, which has been widely studied [4,5,6,7,8,9,10,11]. The traditional method of adding surfactants to form emulsions is effective, but surfactants are expensive and can easily adsorb with rock layers [12]. The alkali–surfactant–polymer system is complex and can be separated by chromatography [13,14,15,16,17]. In recent years, research on amphiphilic polymers has gained more attention. Scholars have confirmed that amphiphilic polymers are beneficial for emulsifying heavy oil. Higher hydrophobic groups can improve the ability to reduce surface tension, while hydrogen bonds and long-chain groups can destroy the asphaltene structure in heavy oil [18,19,20,21,22,23,24,25].

Wang and Tan have developed water-soluble amphiphilic polymers with strong hydrogen bonds, polar functional groups and acrylamide. Including hydrogen bonds and polar groups increases the content of surfactant monomer in the polymer, while the water-soluble hydrophobic long chain enhances the terpolymer’s surface and interface activity. This results in improved emulsification and viscosity reduction capabilities [26]. Guo has synthesized an amphiphilic polymer through free radical polymerization, which can reduce the interfacial tension of oil and water to an ultra-low level. This reduces droplet shrinkage at the interface, promotes oil–water mixing and emulsification and converts high-viscosity heavy oil into a low-viscosity oil-in-water (O/W) emulsion, thereby improving the flow of heavy oil [27]. Tan has synthesized an amphiphilic terpolymer by free radical copolymerization. These copolymers can potentially disrupt the asphaltene structure through the formation of hydrogen bonds, interact with heavy oil through hydrophobic groups and dissociate resin due to strong polar groups. However, due to low surface activity, the oil-in-water emulsion formed is prone to fast self-decomposition and instability, leading to the complete separation of oil and water [28]. Currently, most research on amphiphilic polymer visbreaking agents focuses on preparing polymers containing acrylamide units through free radical polymerization. The synthesis process is relatively complex, and considerations about dehydration, purification and the solubility of the polymer are typically required post-synthesis. Furthermore, acrylamide hydrolyzes easily in water and lacks stability at high temperatures. Therefore, a viscosity reducer with simple synthesis and stable properties is urgently needed.

The aim of this study was to prepare a new amphiphilic viscosity-reducing agent. Currently, there is limited research on acrylate viscosity-reducing agents. This water-based acrylate viscosity reducer can be simply synthesized using emulsion polymerization. The resulting viscosity-reducing agent has excellent stability and uniform dispersion in water, which can be directly added as an emulsion. It can improve the flow of heavy oil and effectively reduce viscosity through emulsion polymerization and the synergistic interaction between acrylate monomers. The polymer’s hydrogen bonds can disrupt the internal structure of heavy oil, and the polymer’s hydrophobic groups and network structure can form a stable emulsion with heavy oil. The new viscosity-reducing agent can avoid delamination and thermal decomposition thanks to its high stability and thermal performance. This new synthesis method offers simplicity, stability, high efficiency and good economic value.

## 2. Materials and Methods

### 2.1. Materials

The ethyl methacrylate (EMA, AR), butyl acrylate (BA, AR), acrylic acid (AA, AR), *N*-hydroxymethyl acrylamide (N-MAM, AR), glycidyl methacrylate (GMA, AR), ammonium persulfate (APS, AR) and sodium bisulfite (SBS, AR) were purchased from Anhui Zesheng Technology Co.(Anqing, China) and polyoxyethylene octylphenol ether-10 (OP-10, AR) was purchased from Beijing Innochem Technology Co. (Beijing, China), Sodium dodecyl sulfate (SDS, AR) was purchased from Shanghai Bidet Pharmaceutical Technology Co. (Shanghai, China), Sodium bicarbonate (NaHCO_3_, AR) was purchased from Tianjin Beichen Fangzheng Reagent Factory (Tianjin, China). M-xylene (AR) and disodium laureth sulfosuccinate (MES, AR) were purchased from Shanghai MackLin Biochemical Technology Co. (Shanghai, China). All chemical reagents could be used directly in our experiments without any purification.

### 2.2. Synthesis of Viscosity Reducer

Butyl acrylate and ethyl methacrylate were selected to increase the solubility of the polymer in heavy oil through nonpolar groups in which ethyl and butyl groups provided more hydrophobic groups. We used *N*-hydroxymethyl acrylamide to provide strong hydrogen bond interactions; acrylic acid was selected to provide hydrogen bonding with the polymer.

The acrylate viscosity reducer was synthesized through semi-continuous emulsion polymerization in a 500 mL four-necked, round-bottomed flask equipped with a mechanical agitator, thermocouple temperature tester, reflux condenser and constant pressure separation funnel. The basic ingredients for preparing acrylate viscosity reducer are shown in Table 1.

The preparation steps for the emulsion are as follows: First, conduct the pre-emulsification process in a 500 mL round-bottomed flask. Add two emulsifiers (SDS and OP-10 in a mass ratio of 4:5) along with deionized water. Insert a stirrer into the round-bottomed flask and stir the mixture at room temperature (25 °C) for 10 min using a mechanical stirrer, until the emulsifier is evenly dispersed in the water. The monomers are then added to the round-bottomed flask in the order of BA, EMA, AA and N-MAM and continue to be stirred for 30 min. Next, take out 1/4 of the pre-emulsion, transfer it to a 500 mL glass four-port flask, connect the condensate, stir and heat until the temperature rises to 50 °C. Next, all NaHCO_3_ aqueous solution (mass 0.02 wt%) is added to the system and the temperature continues to be increased to 60 °C. When that temperature is reached, the initiator APS aqueous solution (mass 2.34 wt%) is added, and the reducing agent SBS aqueous solution (mass 3.66 wt%) is added 30 s later. Adjust the temperature to the required level and add the aqueous SBS solution (0.9 wt% SBS) after 30 s to synthesize the seed emulsion. When the temperature reaches 80 °C, observe whether the mobile phase of the emulsion system is the blue phase. When the system is in the blue phase, the remaining 3/4 pre-emulsion and all APS aqueous solution (mass fraction 3.1 wt %) are added through the constant pressure separation hopper simultaneously. The acceleration of pre-emulsion drop is controlled to be twice the acceleration of APS aqueous solution drop, and the drip is added slowly. After 2 h, the drip is complete. To ensure the reaction is sufficient, it is necessary to maintain the reaction temperature at 80 °C and continue the reaction for another two hours before stopping it. The initiator used in the synthesis process is ammonium persulfate, and the temperature of ammonium persulfate as the initiator is usually 60–90 °C. This study was initiated at temperatures of 60 °C and 80 °C, respectively. Finally, the synthesized emulsion is naturally cooled to room temperature, and ammonia is added to adjust the pH value to neutral or weakly alkaline while stirring. Finally, it is filtered with a 200-mesh screen and named W0 after collection.

In the above synthesis, the preparation process of epoxy acrylate viscosity-reducing agent is similar to that of acrylate viscosity-reducing agent. GMA is introduced into the system during the pre-emulsification stage. The amount of GMA is fixed at 5%, 10% or 15% according to the quality of acrylic monomer. These samples are named Wm (m = 5, 10, 15), representing the epoxy acrylate viscosity reducer prepared with mwt% GMA.

Due to its amphiphilic properties, the polymer can adsorb the oil–water interface with heavy oil and enhance the strength of the oil–water interface film. The introduction of GMA can bring more hydrogen bonds and hydrophobic groups to the polymer, and some epoxy groups may react with the hydroxyl group to form a closer cross-linked structure of the polymer, thus improving the strength of the oil–water interface film.

### 2.3. Preparation of Heavy Oil Emulsion

First, the heavy oil is heated at 150 °C for 20 min, adding 20% thin oil for thinning treatment, and stirring with a stirring rod for 2 min. The benchmark oil sample is prepared, which is named A0. The sample A0 is left to stand until the temperature drops to 80 °C. Combine 4% m-xylene with an aqueous solution of surfactant (0.8 wt% by mass), along with epoxy acrylate viscous-reducing agent W15, and agitate using a stirring rod for a duration of 2 min. The mass ratio of *m*-xylene and surfactant aqueous solution to heavy oil is fixed, and the mass of epoxy acrylate viscosity reducer accounts for 2%, 4%, 6% or 8% of the heavy oil mass. Add the mixture. Finally, stir the mixture with a stirring stick to immediately form an oil-heavy oil emulsion. The obtained samples are represented by A1 (2 wt%), A2 (4 wt%), A3 (6 wt%) and A4 (8 wt%), respectively.

### 2.4. Polymer Characterization

The chemical structure of the synthesized emulsions was analyzed using FT-IR (Nicoleti S50, Thermo Fisher Scientific Co., Waltham, MA, USA) in the wave number range: 4000–400 cm^−1^. A nano laser particle sizer (Nano ZS90, Malvern Panalytical, Nottingham, UK) was used to determine the particle size, polymer dispersibility index and Zeta potential of the emulsions. The sample needs to be diluted with deionized water. Once the sample is diluted to a concentration of 5%, a uniform and stable liquid can be obtained by stirring it magnetically for 10 min. Ultrasound treatment is then applied for an additional 10 min to produce bubbles. A small amount of the liquid is placed in the sample pool for analysis, and each sample is tested three times at a temperature of 25 °C. After the particle size has been determined using this method, the same preparation process is followed for the Zeta potential testing: the sample is diluted to 5%, stirred, subjected to ultrasound and then transferred to the sample pool. This test is also conducted three times at room temperature, which is maintained at 25 °C.

### 2.5. Thermal Properties’ Analysis

The thermal stability of the viscosity reducer is tested by TGA (Mettler Toledo Co., Zurich, Switzerland). The test conditions are set as follows: the starting temperature of the test temperature is 20 °C, the end temperature is 800 °C, the heating rate is 10 °C/min and the flow rate of N2 is 30 mL/min.

### 2.6. Evaluation of the Viscosity Reduction Effect

The viscosity of the dispersing medium in the emulsion was determined by an Anthopar MCR 301 rheometer concentric cylinder system (measuring cup radius 14.466 mm, rotor radius 13.330 mm, Anton Paar GmbH, Graz, Austria), and we set the shear rate of the test to 0.01 s^−1^ to 100 s^−1^. The test temperature was 50 °C.

The viscosity reduction rate (VRR) was used to reflect the viscosity reduction ability of the polymer to heavy oil. The viscosity reduction rate is shown by Formula (1):

(1) VRR (%) = (η0 − η1) × 100/η0

where η0 signifies the baseline viscosity of heavy oil before undergoing viscosity reduction, measuring 1596 mPa·s. η1 indicates the viscosity achieved in the O/W emulsion after completing the viscosity reduction procedure.

## 3. Results and Discussion

### 3.1. Reaction Mechanism of Epoxy Acrylate Viscosity Reducer

**Figure 1:**
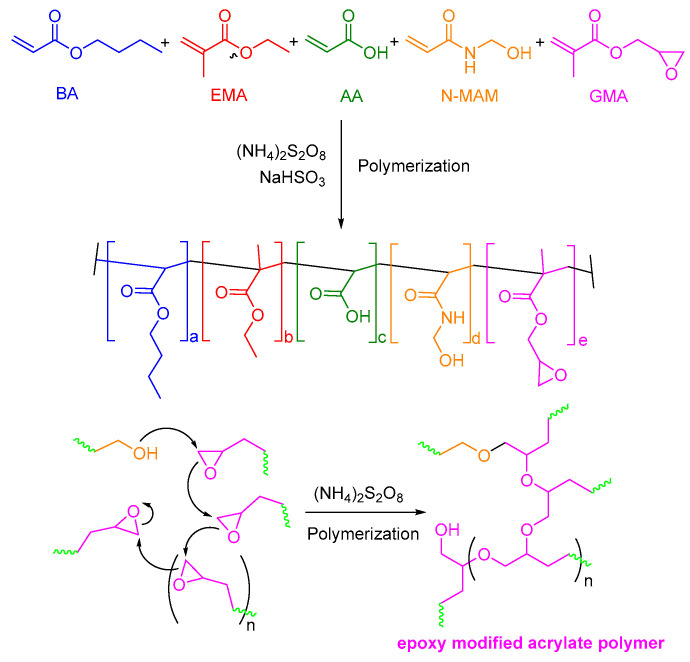
Synthesis of epoxy acrylate polymer.

The reaction mechanism of an epoxy acrylate viscosity reducer in the synthesis process can be shown in Figure 1. The synthesis process is carried out in an oxidation–reduction initiation system, where the carbon–carbon double bond (C=C) in the acrylic monomer undergoes radical polymerization, resulting in the formation of a polyacrylate chain that contains hydrogen bonds and hydrophobic groups. With the addition of the epoxy monomer GMA, carbon–carbon double bonds in epoxy monomer GMA participate in radical polymerization, introducing the epoxy group segment into the main chain of the polyacrylate and forming copolymers containing epoxy in the side chain. During the polymerization process, some epoxides in GMA may undergo epoxy-ring-opening reactions with hydroxyl groups to form ether bonds.

**Figure 2:**
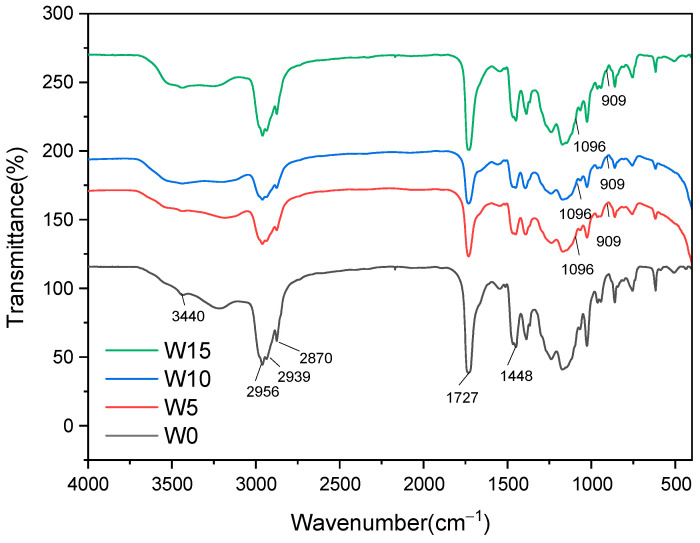
The FTIR spectra of the viscosity reducer include the FTIR spectra of GMA (W0), 5 wt% GMA (W5), 10 wt% GMA (W10) and 15 wt% GMA (W15).

We next sought to verify the successful introduction of GMA in a pure acrylic viscosity reducer. Figure 2 illustrates the FTIR spectra of W0, W5, W10 and W15. In the FTIR spectra of pure acrylic viscosity reducer W0, -CH_3_ and -CH_2_- have stretching vibration absorption peaks at 2956 cm^−1^ and 2870 cm^−1^, and in the FTIR spectra of pure acrylic viscosizer W0, -CH_3_ and -CH_2_- have bending vibration absorption peaks at 1448 cm^−1^, -NH and -OH have tensile absorption peaks at 3440 cm^−1^ and C=O have tensile absorption peaks at 1727 cm^−1^. In the FTIR diagram of GMA (W5, W10, W15), it can be seen that the tensile vibration at 909 cm^−1^ is the epoxy group, and the absorption peak at 1096 cm^−1^ is the C-O-C tensile vibration, which may be due to the ring-opening of some epoxy groups, indicating that GMA has successfully introduced the polyacrylate molecular chain. Due to the addition of GMA, which introduces epoxy groups that require more heat to decompose, some of these epoxy groups can react with -OH, enhancing the spatial network structure of the polymer and further improving thermal stability.

### 3.2. Effect of GMA Dosage on the Stability of Viscosity Reducer

**Figure 3:**
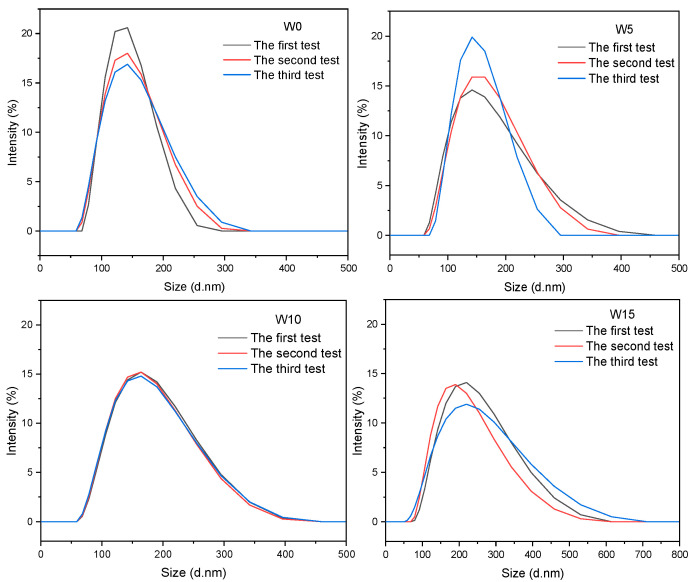
Particle size distribution profiles of epoxy acrylate viscosity reducers with GMA contents of 0 wt% (W0), 5 wt% (W5), 10 wt% (W10) and 15 wt% (W15), respectively.

The particle size distribution curves of epoxy acrylate viscosity reducers with GMA contents of 0 wt%, 5 wt%, 10 wt% and 15 wt% are shown in Figure 3. The particle size distribution of the synthesized epoxy acrylate viscosity reducers appears relatively uniform, unimodal and normally distributed, as depicted in the figure. The polydispersity index (PDI) is utilized to characterize the molecular weight distribution of polymers. A higher PDI indicates a broader molecular weight distribution, while a lower PDI signifies a more uniform molecular weight distribution. The particle size distribution was narrow when the GMA content was 0 wt%~10 wt% (50~400 nm, PDI 0.066~0.119), while the particle size distribution was wide when the GMA content was 15 wt% (50~700 nm, PDI 0.173). This finding aligns with the particle size dispersion index presented in Table 2. As this shows, when the addition of GMA was 0% or when it was 5% or 10%, the emulsion particle size of the epoxy acrylate viscosity reducer synthesized by GMA changed little. When the GMA content was 15 wt%, the particle size of the emulsion increased. This was due to the large amount of GMA and the introduction of more acrylate chains and epoxy groups, which enhanced the structure of the polymer. Therefore, the particle size of the W15 viscosifier emulsion was relatively large. The Zeta potential of the synthesized epoxy acrylate viscosity reducer was in the range of ±40~±60 mV, and we found that the viscosity reducer had strong stability and did not easily aggregate. Both the particle size and Zeta potential are important indicators of emulsion stability. Generally, smaller particle sizes correspond to larger absolute Zeta potentials, which indicates a more stable emulsion. An emulsion with an absolute Zeta potential value between 40 and 60 has good stability, making it less likely to experience issues such as precipitation, delamination or condensation. As the amount of GMA increases, the spatial density among the polymers increases, leading to an enhanced polymer structure. This results in a gradual increase in polymer particle size; however, the variation is minimal, and all samples demonstrate good stability.

### 3.3. Thermal Stability Measurement

**Figure 4:**
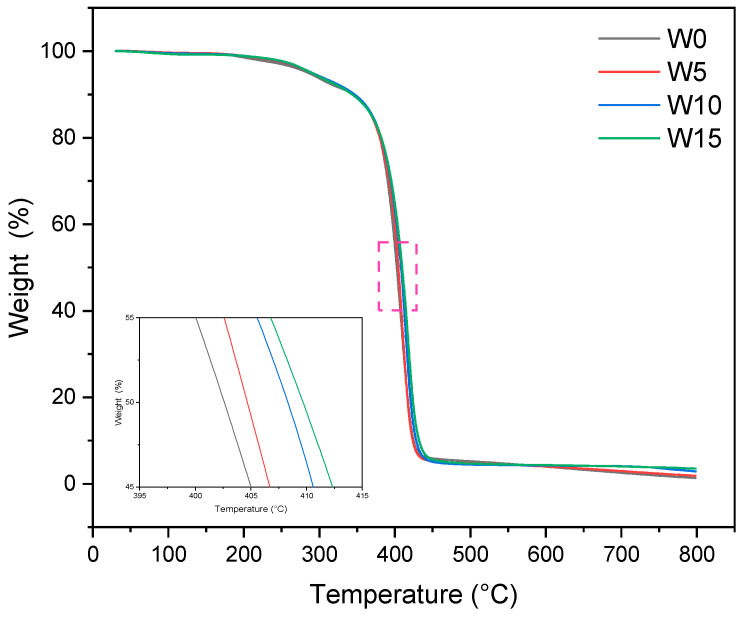
TG curves of epoxy acrylate polymers with 0 wt% (W0), 5 wt% (W5), 10 wt% (W10) and 15 wt% (W15) GMA contents, respectively.

The thermal performance of W0–W15 was tested by the thermogravimetric analyzer. The thermal behavior of the acrylate viscosity reducer is shown by the TG curve in Figure 4.

The thermal degradation behavior of W0–W15 under nitrogen at 20–800 °C is shown in Figure 4. All samples exhibited similar degradation behavior. The weight loss at 100–200 °C is due to the removal of unremoved water molecules, while the weight reduction at 350–480 °C is attributed to the decomposition of macromolecular chains. The initial decomposition temperatures were 366 °C, 368 °C, 368 °C and 370 °C, and the final decomposition temperatures were 450 °C, 475 °C, 493 °C and 517 °C, respectively. With the increase in GMA content, the polymer’s thermal decomposition temperature and thermal stability increased in a certain range. Compared with W15, the final thermal degradation temperature of W0 increased from 450 °C to 517 °C. This may be due to the introduction of epoxy groups by adding GMA, which requires more heat to decompose, and some of these epoxy groups can react with -OH, enhancing the spatial network structure of the polymer and further improving thermal stability. Through the test, it can be seen that the thermal stability increases with the increase in GMA content. Among them, W15 has the best thermal stability, which is better than those of the other three polymers. In addition, with the addition of GMA, the thermal degradation of the resin is delayed, as confirmed by the insertion curve in Figure 4. In the specific research range, the delayed thermal degradation showed an increasing trend with the gradual increase in GMA content. The addition of glycidyl methacrylate (GMA) introduces epoxides that require higher temperatures to decompose. Some of these epoxides can react with hydroxyl groups (-OH), which enhances the spatial network structure of the polymer and further improves its thermal stability. When the GMA content reached 15 wt%, the maximum weight loss temperature increased by approximately 12 °C compared to the sample without GMA. Among the synthetic polymers, W15 polymer introduced the most GMA and showed the best thermal stability. Therefore, compared with other synthetic polymers, it has a significant advantage in the viscosity reduction of heavy oil and is an ideal choice.

### 3.4. Heavy Oil Viscosity Reduction Performance of Polymer

The selected W15 epoxy acrylate viscosity-reducing agent can better meet the requirements of viscosity reduction of heavy oil. It improves fluidity and reduces the viscosity of heavy oil by wrapping heavy oil in a polymer aqueous solution to form an O/W emulsion. Additionally, W15 can be mixed with organic solvents to produce a synergistic effect. Xylene essentially changes the internal structure and properties of heavy oil, redistributes heavy oil molecules through its special structure, disassembles the macromolecular structure and destroys the strong adhesion of heavy oil [29]. When m-xylene auxiliary viscosity-reducing agent is added to the epoxy acrylate viscosity-reducing agent, it can improve the viscosity-reducing effect. Since the epoxy acrylate viscous-reducing agent is dispersed in a water system and is not miscible with m-xylene, a small amount of surfactant aqueous solution is added to combine the two for the viscosity reduction of heavy oil.

**Figure 5:**
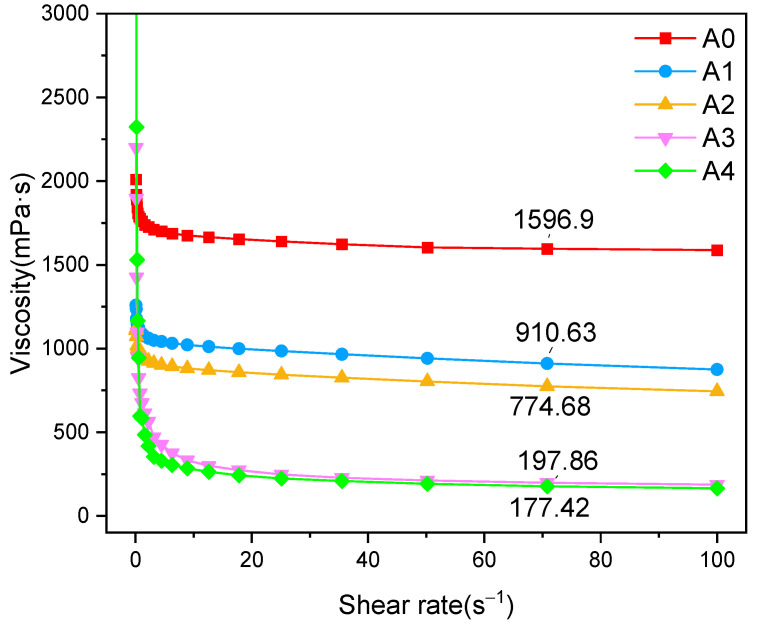
Viscosity changes of W15-type epoxy acrylate viscosity-reducing agent with different concentrations and at different shear rates.

In Figure 5, the viscosity changes of W15 epoxy acrylate viscosity-reducing agent with different concentrations are shown at different shear rates and a test temperature of 50 °C. As can be seen from the figure, with the gradual increase in shear rate, the viscosity of A0–A4 decreases rapidly. According to the literature, the viscosity of heavy oil decreases as the shear rate increases due to reduced entanglement and bonding between the heavy oil molecules or emulsions [30,31,32,33,34,35,36,37,38,39,40]. For instance, the viscosity of A0 is 1596.9 mPa·s at a shear rate of 70 s^−1^. Additionally, the figure demonstrates that with the addition of a viscosity reducer, the viscosity of heavy oil decreases significantly. For instance, after adding A1 and A2, the viscosity of heavy oil decreases from 1596.9 mPa·s to 910.63 mPa·s and 774.68 mPa·s, respectively. Furthermore, as the viscosity-reducing agent content increases, the viscosity of heavy oil decreases more noticeably. For example, after adding A3, the viscosity of heavy oil decreases to 197.86 mPa·s, and after adding A4, the viscosity decreases to 177.42 mPa·s. According to the viscosity test results in Figure 5, it can be concluded that the addition of W15 viscosity-reducing agent brings about a viscosity-reducing effect. Especially after the addition of 6% or 8% viscosity reduction agent, a significant viscosity reduction effect is produced.

**Figure 6:**
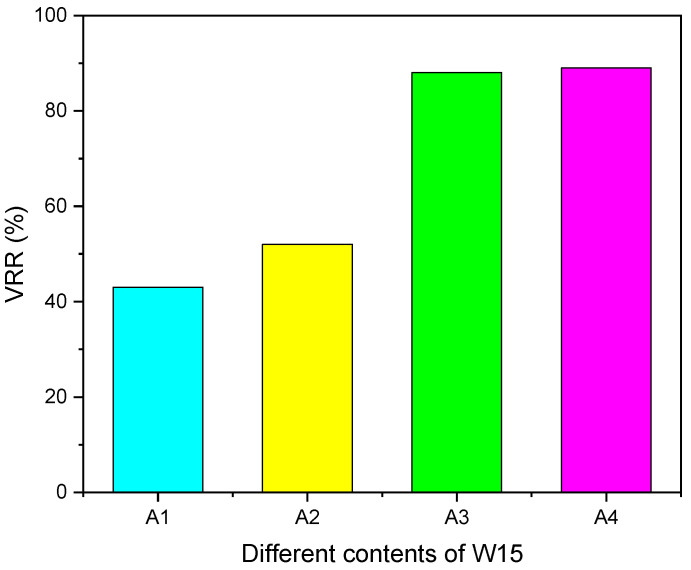
Viscosity reduction of heavy oil with W15 viscosity reduction agent contents of 2 wt% (A1), 4 wt% (A2), 6 wt% (A3) and 8 wt% (A4), respectively.

**Figure 7:**
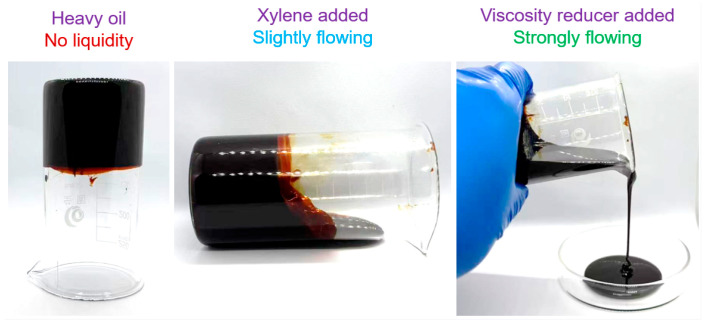
Effect of adding a viscosity reducer on the fluidity of heavy oil.

Figure 6 shows the viscosity reduction rate of A0–A4 at a 70 s^−1^ shear rate. VRR was calculated by the viscosity reduction formula, and η0 was 1596.9 mPa·s. With the increase in W15 viscosity reducer content, the viscosity reduction rate gradually increases. Specifically, the viscosity reduction rate for A1 reaches 43%, while for A2, it is 52%. When 6% and 8% W15 viscosity reducers are added, the viscosity reduction rates for A3 and A4 increase significantly, reaching 88% and 89%, respectively. The effect of adding viscosity reducers on the flow rate of heavy oil is illustrated in Figure 7. When the content of W15 is 2–8%, the viscosity reduction rate can reach 43–89%. The results show that the epoxy acrylate viscosity reducer has a good viscosity reduction effect. When a small amount of m-xylene destroys the structure of heavy oil, the hydrophobic groups in W15 adsorb at the oil–water interface, and the polar groups in W15 increase the polymer’s solubility in heavy oil. In the meantime, the hydroxyl and amide groups in the polymer form strong hydrogen bonds with the asphaltenes in the internal structure of the heavy oil, thus reducing the viscosity of the heavy oil. Combined with the test results of viscosity and viscosity reduction in Figure 5 and Figure 6, it can be seen that the synthesized W15 epoxy acrylate viscosity reduction agent has a satisfactory effect.

## 4. Conclusions

In this study, a viscosity-reducing agent was developed using emulsion polymerization with butyl acrylate and methyl methacrylate as the main monomers, along with the addition of acrylic acid and *N*-hydroxymethyl acrylamide. A series of viscosity-reducing agents were created by incorporating different amounts of glycidyl methacrylate (GMA) monomer. Analysis of the particle size and Zeta potential structure indicated the excellent stability of the synthesized viscosity reducer. Furthermore, the thermogravimetric analysis revealed a significantly improved thermal stability of the GMA-modified resin, with W15 exhibiting the best thermal stability among the four viscosity-reducing agents.

Viscosity tests and viscosity reduction rates of heavy oil showed a noticeable decrease in viscosity and a substantial increase in viscosity reduction rate with the higher W15 content. The addition of 2% W15 epoxy acrylate leads to a viscosity reduction rate of 43% in heavy oil, while a 4% addition results in a 52% reduction. Although the effect is relatively modest with these lower percentages, adding 6% W15 epoxy acrylate achieves an impressive 88% viscosity reduction. Furthermore, with an 8% addition, the viscosity reduction rate reaches 89%, showcasing an exceptional performance.

This study successfully synthesized a novel epoxy acrylate viscosity-reducing agent using a straightforward synthesis method. The polymer’s hydrophobic groups adsorbed at the oil–water interface, while the polar groups enhanced solubility in heavy oil. Additionally, the hydroxyl and amide groups in the polymer formed strong hydrogen bonds with the asphaltenes in the heavy oil, leading to reduced viscosity. This viscosity reducer exhibits excellent stability, cost-effectiveness and remarkable viscosity reduction effects. Its stability and dispersion performance within the system minimize the likelihood of precipitation, ensuring an impressive long-term storage performance. Currently, it is specifically applicable to the synthesis and viscosity reduction of acrylates. The balance of hydrophilic and hydrophobic groups in the viscosity reducer can be further adjusted and modified. More research is needed to explore the hydrophilic and hydrophobic balance of the new viscosity reducer in various oils. This study holds considerable research value and is important for the development of amphiphilic polymer viscosity-reducing agents.

## Figures and Tables

**Table 1:** Basic raw materials for Sample W0.

Component	Amount (wt%) ^a^	Component	Amount (wt%) ^a^
EMA	15	SDS, OP-10	1.8
BA	13.5	APS	0.6
AA	0.6	SBS	0.19
N-MAM	0.9	Deionized water	67.41

^a^: Percentage of the total.

**Table 2:** Effect of GMA dosage on the stability of viscosity reducer.

Sample	Appearance of Latex	Z-Average Particle Size (d. nm)	Polydispersity Index (PDI)	Zeta Potential (mV)
W0	milky white, intense blue light	132	0.066	−43
W5	milky white, intense blue light	142	0.09	−42
W10	milky white, intense blue light	150	0.119	−42
W15	milky white, low blue light	187	0.173	−40

## Data Availability

Data are contained within the article.

## Abbreviations

The following abbreviations are used in this manuscript:

BA	butyl acrylate
EMA	ethyl methacrylate
AA	acrylic acid
N-MAM	*N*-hydroxymethylacrylamide
GMA	glycidyl methacrylate
FTIR	Fourier transform infrared spectroscopy
TG	thermogravimetric
O/W	oil-in-water
VRR	viscosity reduction rate
